# Experimental and numerical study on photocatalytic activity of the ZnO nanorods/CuO composite film

**DOI:** 10.1038/s41598-020-64784-w

**Published:** 2020-05-08

**Authors:** Dung T. Nguyen, Minh Duc Tran, Thanh Van Hoang, Duc Thien Trinh, Duc Thang Pham, Dinh Lam Nguyen

**Affiliations:** 1 Faculty of Engineering Physics and Nanotechnology, VNU University of Engineering and Technology, Vietnam National University, 144 Xuan Thuy Road, Cau Giay District, Hanoi 100000 Vietnam; 2 Faculty of Physics, Hanoi National University of Education, 136 Xuan Thuy Road, Cau Giay District, Hanoi 100000 Vietnam

**Keywords:** Structural properties, Environmental chemistry, Materials for energy and catalysis

## Abstract

The photocatalytic activity of the ZnO NRs/CuO composite film was investigated by using both experimental and numerical methods. The ZnO NRs/CuO composite film exhibits significantly enlarge absorption range to visible-light and suppress the recombination rate of the photogenerated electron-hole pairs, which can be well utilized as a photocatalyst. The ZnO NRs/CuO composite film also presents good stability, and reusability, and durability for photo-decomposition purpose. The optimal ZnO NRs/CuO composite film contains 1μ-thick of CuO film and 10 nm-thick of ZnO NRs film. The donor concentration in ZnO NRs film should be lower than 10^16^ cm^−3^. The short circuit current density of the optimal composite film is 25.8 mA/cm^2^ resulting in the calculated pseudo-order rate constant of 1.85 s^−1^. The enhancement in degradation efficiency of this composite film is attributed to the inner electric field and large effective surface area of ZnO NRs film.

## Introduction

The treatment of organic pollutants in waste-water by semiconductor has been a promising method among advanced oxidation processes^1–5^. Photocatalytic technology plays as an environmental-friendly and potential way to degrade organic pollutants into nontoxic inorganic compounds without generating secondary contamination. Under light irradiation, semiconductors can generate electron-hole pairs that are highly reactive and can participate in a series of a redox reaction to produce efficient superoxide radicals (^*^O_2_^−^, HO_2_^*^) for pollution decomposition^2,5^. Among several semiconductors such as V_2_O_5_^6^, Fe_2_O_3_^7^, Cu_2_O^8^, TiO_2_^9^, and BiVO_4_^10^, the pure ZnO which has been widely used as a photocatalyst in the photocatalytic degradation of organic pollutants in aqueous solutions because of its nontoxicity, low cost, high redox potential^11,12^. However, ZnO was shown as a pristine semiconductor with wide bandgap (E_g_ = 3.37 eV), which operates well under ultraviolet (UV) irradiation, but poorly under visible light^13,14^ while the UV light accounts for only a small proportion (4%) of solar irradiation in comparison to the visible light percentage (43%). Therefore, most portions of sunlight cannot be absorbed by the pure ZnO and the improvement of degradation efficiency under the catalyst of ZnO was severely restricted by solely modifying its size, morphology, and structures^11,15–17^. Furthermore, the ZnO exhibits direct pathways for charge carrier transfer, which causes fast recombination of generated electron-hole pairs^16^. These are unfavorable properties of ZnO preventing it from the high performance of photocatalysis. Therefore, it is urgent to expand visible absorption range and suppress electron-hole pair recombination by fabricating ZnO-based composite photocatalysts which would contribute to the enhancement of photocatalytic activity.

Recently, ZnO was studied in the form of ZnO-based heterojunctions of other p-type narrow-bandgap semiconductors such as Si, CuO, Cu_2_O, NiO, and CdS, which is considered as a promising solution to solve the above disadvantages^1,2,18–26^. Photogenerated electrons and holes can migrate to related counterparts by an inner electric field created by the heterojunction, leading to an enhancement in the charge separation, which contributes to the improvement of photocatalytic activity due to the more generated charge carriers taken part in the redox reactions of the photocatalytic process^18,27^. In addition, the heterojunction between wide-bandgap ZnO and narrow bandgap semiconductors expands UV-light photoresponse of ZnO to UV-visible region, as a result of the enhancement of the light absorption ability. Among all p-type narrow bandgap semiconductors, copper oxide (CuO) has turned out to be one of the excellent candidates for creating p-n heterojunctions with ZnO because of its narrow-bandgap (1.35 eV), high optical absorption, nontoxicity, and low electrical resistance values^28–31^. However, ZnO/CuO structures were usually fabricated in the form of nanopowders, nanowires, or flower-like three-dimension^32,33^, which have disadvantages in separating and recovering catalytic materials after the water treatment process. Therefore, a film of ZnO/CuO structure should be investigated to solve the above problems. Furthermore, CuO nanomaterial is usually synthesized on the surface of the ZnO nanostructure^2,34^. That means the CuO material will cover a part of surface of the ZnO nanostructure, which leads to a decline in the photocatalytic efficiency of a synthesized nanostructure. To specify, when a composite of CuO and ZnO was exposed to the light source, most of the high energy photons (UV light) of the incident light will reach and be absorbed by CuO materials first, as a result, the number of photons in UV regions approach and are absorbed by ZnO will be reduced. Therefore, further investigation of the ZnO-based heterojunction of p-type CuO composite films (ZnO NRs/CuO) with CuO doing not cover the surface of ZnO NRs in photocatalytic activity is still required in detail.

In this work, the ZnO NRs/CuO composite film were fabricated on a glass substrate by sputtering, thermal annealing, spin coating and simple hydrothermal methods. For comparison, CuO film, ZnO NRs, and CuO/ZnO NRs composite films were also fabricated on glass substrates by the same methods. The photocatalytic activities of all fabricated samples have been investigated. The results indicated that the ZnO NRs/CuO composite films show the highest photocatalytic activities compared to other samples. The highest photocatalytic activities of the ZnO NRs/CuO composite film would be ascribed to the extension of the optical absorption range and the efficient separation of photogenerated electron-hole pairs. These results were also confirmed by numerical study using SCAPS device simulation. Based on the simulation program, characteristics of the ZnO NRs/CuO composite film such as the thickness of the CuO, ZnO NRs films, and donor density in the ZnO NRs film were also investigated to find out the optimal structure. Furthermore, the photocatalytic activity of the ZnO NRs/CuO composite film also depends on the illuminated side.

## Results and Discussion

### SEM analysis

**Figure 1:**
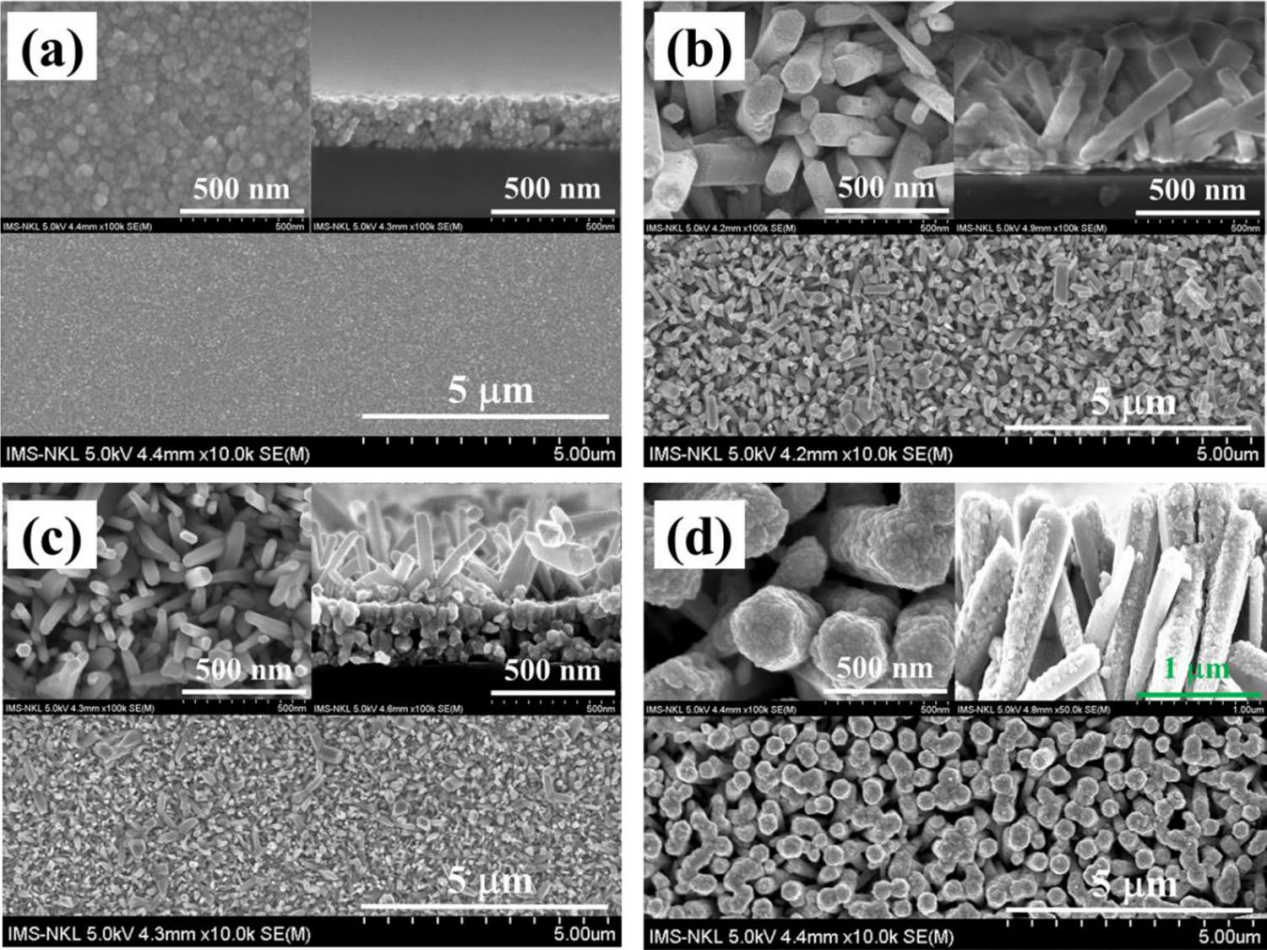
Top- and side-view SEM images of (**a**) CuO film and (**b**) ZnO NRs film, (**c**) ZnO NRs/CuO composite film, (**d**) CuO/ZnO NRs composite film.

Figure 1 shows the SEM morphology evolution of the fabricated samples. 300 nm thick of CuO nanosheet film formed from CuO nanoparticles and 450 nm thick of ZnO nanorods film on the glass substrates can be clearly observed in Fig. 1(a,b), respectively. The ZnO NR diameters which are grown on the glass substrate are in range from 50 – 70 nm and well-aligned. These are also similar for the ZnO NR structure grown on the CuO nanosheet film as shown in Fig. 1(c). This result indicated that the ZnO NRs/CuO composite film was well constructed via the processes in the experimental part. The inverted CuO/ZnO NRs composite film structure was also fabricated for comparison purpose and shown in Fig. 1(d). In this figure, the CuO nanoparticles were adhered to the ZnO NRs, which also create contact between CuO and ZnO NRs according to a p-n heterojunction formed.

### XRD analysis

**Figure 2:**
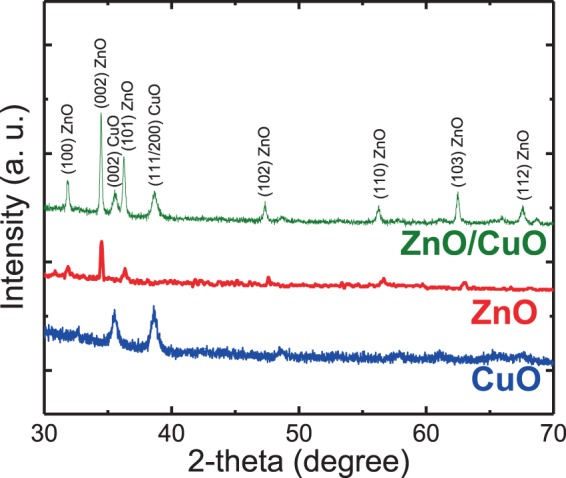
XRD patterns of the CuO, ZnO film and ZnO NRs/CuO composite film.

The crystalline structures of the CuO film, ZnO NRs, and the ZnO NRs/CuO composite film were investigated by the XRD analysis as shown in Fig. 2. The diffraction peaks of all samples are well defined, revealing the good crystallinity of the fabricated samples, and no peak of other phase and impurity is detected.

In the diffraction pattern of ZnO NRs, it indicates that the crystalline structure of the ZnO NRs film synthesized by the hydrothermal method is in a hexagonal wurtzite structure^4^. Beside that the much higher intensity of the (002) diffraction peaks indicates the excellent c-axis orientation of this ZnO NRs film which is consistent with the observation in the SEM images.

In the diffraction pattern of the CuO film, two peaks at 2θ of 35.4^o^ (0 0 2) and 38.1^o^ (2 0 0) are clearly observed which are well assigned to the presence of the CuO phase. The XRD peaks of the CuO film show a large full width at half maximum (FWHM) of the peaks and low intensity, which most probably is due to the small grain size of CuO nanoparticles, that construct the CuO film. The absence of any other copper species peaks in the XRD patterns proves that copper nanoparticles after the thermal annealing process are completely converted to copper oxide.

In the diffraction pattern of the ZnO NRs/CuO composite film, all of the peaks related to the ZnO and CuO crystalline structures can be easily observed. This result is another evidence to confirm the forming of the ZnO NRs/CuO composite film.

### Optical absorption analysis

**Figure 3:**
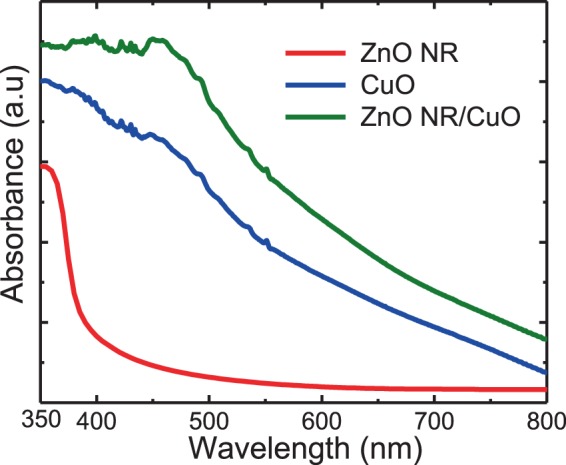
Absorption spectra of the CuO, ZnO film and ZnO NRs/CuO composite film.

Figure 3 shows the optical absorption spectra of the CuO film, ZnO NRs film, ZnO NRs/CuO composite films. The ZnO NRs film shows a band gap energy at around 375 nm (~3.3 eV). The CuO film shows a high and broad range of light absorption up to 800 nm. Meanwhile, the ZnO NRs/CuO composite film behaviors not only a broad range absorption but also the highest optical absorption in comparison to that of the ZnO NRs film and the CuO film. This phenomenon can be explained by attributions of the high surface roughness of the ZnO NRs film in the ZnO NRs/CuO composite film, which can reduce the optical reflection at the surface of the composite film^13^.

### Photocatalytic activity

**Figure 4:**
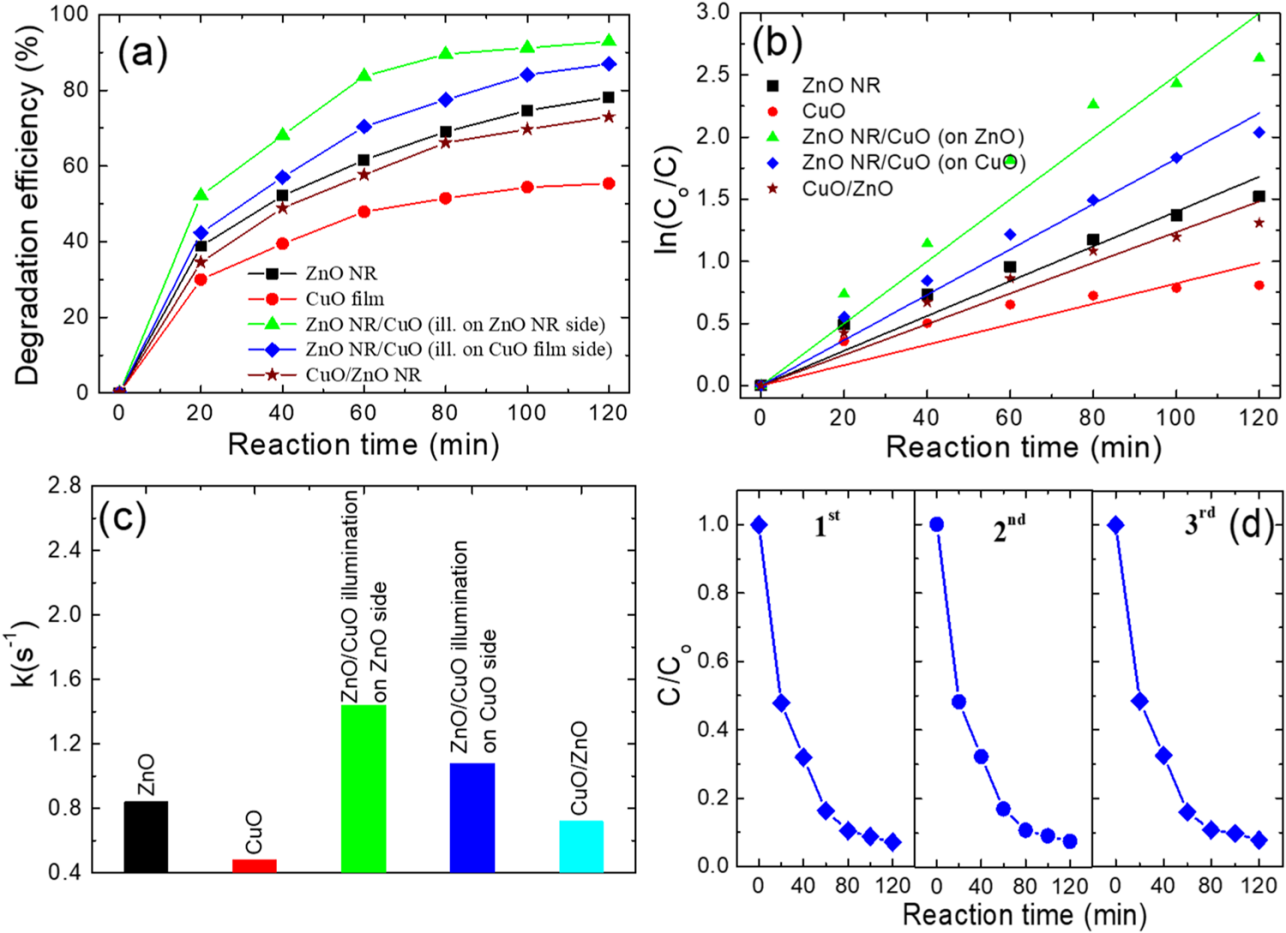
(**a**) Photodegradation of RhB under Xenon lamp (**b**) the first-order kinetic plot for RhB photodegredation, (**c**) pseudo-order rate constant, and (**d**) recycling photodegradation of fabricated samples.

The photocatalytic activities of the fabricated samples were examined by the rate of degradation of RhB contamination under the irradiation of the Xenon lamp, which were shown in Fig. 4. After 30 min of maintaining in the dark, the change in concentration of RhB solution was insignificant, which indicates that the adsorption of RhB in the fabricated samples is negligible. During the photodegradation investigation, the variation of RhB concentration was analyzed by the RhB absorption peak intensity at a wavelength of 554 nm. The mechanism of photocatalytic activity of samples was well described in our previous publication^18^. The obtained results were incorporated in the Eq. (1) to calculate the degradation efficiencies for different samples which are shown in Fig. 4(a). After 120 min of illumination on the fabricated samples side, the degradation efficiencies of the CuO film, ZnO NRs film, and ZnO NRs/CuO composite films are 55, 78, and 93%, respectively. This result indicated that the degradation efficiency of ZnO material can be enhanced by incorporation with the CuO material. Furthermore, the degradation efficiency of the inverted structure (CuO/ZnO NRs composite film) is 73% after 120 min of illumination, which is about 20% lower than that of the ZnO NRs/CuO composite film. The lower in the degradation efficiency of the inverted structure could be explained by the absorbing UV light of ZnO when its surface is covered by CuO material. To specify, when incident light from xenon lamb come to the inverted structure (CuO/ZnO NRs composite film), high energy photons from UV light will approach CuO materials first and be absorbed mostly by CuO, which leads to the smaller amount of UV light will approach and be absorbed by ZnO NRs in comparison with that of ZnO NRs/CuO structure. As a result, the light absorption efficiency of the inverted structure is reduced. To find more evidence to demonstrate that photon absorption is affected by the cover of CuO materials on the ZnO NRs, the comparison of the photodegradation efficiency of the ZnO NRs/CuO composite film under light illumination on different sides (the ZnO NRs side and the CuO side (through glass substrate side)) was investigated. After 120 min of illumination, the degradation efficiencies of the ZnO NR/CuO composite film with ZnO NRs side and CuO side illumination are 93% and 87%, respectively. The degradation efficiency of ZnO NRs/CuO composite film under illumination on the CuO side is about 6% lower than that under illumination on the ZnO NRs side and 14% higher than the efficiency of the inverted structure (CuO/ZnO NRs composite film). The lower in photocatalytic activity of the ZnO NR/CuO composite film when it is illuminated on the CuO side confirms that the photon absorption of ZnO NRs film is reduced when its surface was covered by CuO. In this case, ZnO NRs which are covered by CuO materials in the structure only create the heterojunction to reduce the photogenerated electron-hole pairs recombination. Furthermore, when incident light illuminates on the top surface of ZnO NRs/CuO composite film, ZnO NRs are not covered by CuO materials like the inverted CuO/ZnO NRs structure. Therefore, ZnO NRs will effectively absorb UV light first and the remaining UV light and visible light will approach to CuO layer and be absorbed by that CuO materials, as a result of photon absorption increased and higher degradation efficiency obtained in comparison to that of the inverted CuO/ZnO NRs structure.

The first-order kinetics of RhB photodegradation were calculated and depicted in Fig. 4(b). The pseudo-order rate constant (k) was determined from the slope of the line and shown in Fig. 4(c). The result indicated that the k value of the ZnO NRs/CuO composite film in degrading RhB concentration is approximately 2 times higher than that of the inverted CuO/ZnO NRs structure. In addition, the cycling photodegradation in Fig. 4(d) shows that the ZnO NRs/CuO composite film maintains good degradation efficiency for RhB contamination after three cycling experiments. This result indicates that the ZnO NRs/CuO composite film is a high photostable and a reusable photocatalyst.

### Simulation of the ZnO/CuO heterojunction

**Figure 5:**
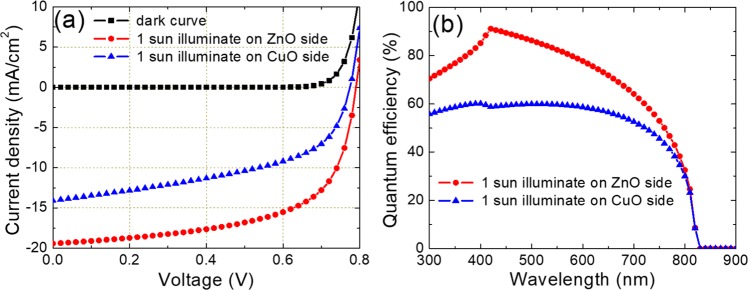
(**a**) J-V curves and (**b**) Quantum efficiencies of the ZnO/CuO heterojunction with illumination on different sides.

The J-V characteristics for the ZnO/CuO heterojunction related to the non-illumination, illumination on the ZnO side, and the CuO side were shown in Fig. 5(a). Under 1 sun illumination, the short circuit current density when the CuO side is shined is 14.1 mA/cm^2^ and is 19.4 mA/cm^2^ if the ZnO side is shined. The short circuit current density when the ZnO NRs side is shined is approximately 1.38 times higher compared to case shining on the CuO side shining. That means the degradation efficiency of ZnO/CnO composite film under illumination on the ZnO side can be 1.38 times faster than that under illumination on the CuO side. This result is consistent with the experimental pseudo-order rate constant in both cases as shown in Fig. 4(b). Furthermore, the k value of the ZnO NRs/CuO composite film is 2 times higher than that of the inverted CuO/ZnO NRs structure. These results demonstrated that the effective surface area of ZnO NR film might contribute up to 62% of the improvement of the degradation efficiency of the ZnO NR/CuO composite film.

Figure 5(b) shows the quantum efficiency of the ZnO/CuO composite heterojunction when it is shined on each sides. The quantum efficiency when light shines on the ZnO NRs side is much higher than that case illumination on the CuO side, especially at wavelengths of around 400 nm. In this structure, when the ZnO NRs side is illuminated, the UV light with wavelength up to 375 nm will be absorbed by the ZnO NRs film. The light with wavelength longer than 375 nm will go through the ZnO NRs film and reach to the CuO film at the depletion area, and will be absorbed by the CuO film. The photogenerated electron-hole pairs at this position will be easily separated by the inner electric field and transferred to each electrode parts. In another case, when illumination on the CuO side, most incident light is absorbed by the CuO film at the position near the electrode part. Therefore, photogenerated electron-hole pair at this position is weakly controlled by the inner electric field and easily recombination resulting in lower photon induced current efficiency^13^.

Based on this SCAPS program, the influences of the thickness of CuO and ZnO NRs films, and donor concentration in ZnO NRs film on the opto-electronic of the ZnO NRs/CuO heterojunction were also investigated to find out the optimal structure (see Supplementary Information). The results indicated that the optimal structure could be obtained with 1μ-thick of CuO film and 10 nm-thick and low donor concentration (under 10^16^ cm^-3^) of ZnO NRs film. This result indicated that, using the ZnO NR/CuO composite film for photocatalyst application, the dopant in ZnO film lacks efficient.

## Conclusions

The photocatalytic activity of the ZnO NR/CuO composite film was investigated by using both experimental and numerical methods. The results indicated that the ZnO NR/CuO composite film can be well utilized as a photocatalyst. The optimal ZnO NR/CuO composite film contains 1μ-thick of CuO film and 10 nm-thick and low donor concentration (under 10^16^ cm^−3^) of ZnO NRs film. The short circuit current density of the optimal composite film is 25.8 mA/cm^2^ resulting in the calculated pseudo-order rate constant of 1.85 s^−1^. The enhancement in degradation efficiency of this composite film is attributed to the inner electric field and large effective surface area of ZnO NRs film.

## Methods

### Materials

Zinc nitrate hexahydrate (Zn(NO_3_)_2_·6H_2_O, ≥99%), hexamethylenetetramine (C_6_H_12_N_4_), NaOH, and methanol were all purchased from Sigma-Aldrich. All reagents were analytic reagent grade and utilized without further purification. Glass substrates with a size of 2 cm ×2 cm ×0.1 cm were used in this work to support the ZnO NRs/CuO composite film.

### Preparation of ZnO NRs/CuO composite film on a glass substrate

Firstly, CuO film was synthesized on a glass substrate using the sputtering and thermal annealing methods. To specify, the clean glass substrate was put in a vacuum chamber and then sputtered of copper for 30 min and under the pressure of 2.6×10^−3^ Torr to create a thin copper film with a thickness of approximately 180 nm. After the sputtering process, the copper film on the glass substrate was annealed at 500 ^o^C for 3 h to obtain the CuO film which was well adhered to the glass surface. The thickness of the fabricated CuO film is approximately 300 nm as shown in Fig. 1(a).

Secondly, ZnO NRs film was grown on the CuO film by the hydrothermal method. Particularly, the ZnO nanoparticles seed layer was synthesized on the CuO nanosheet film by uniformly spin coating at speed of 3000 rpm for 2 min following by thermal annealing at 500 ^o^C for 1 h. Then, ZnO NRs were grown over the ZnO seed/CuO film under the hydrothermal process. In detail, a growth solution which contains 50 mL of 20 mM zinc nitrate hexahydrate and 20 mM hexamethylenetetramine was transferred into an autoclave. Afterward, the glass which was covered by a CuO film and coated with a ZnO seed film was immersed into the growth solution and baked at 100 ^o^C for 2 h. After 2 hours of baking, the autoclave was allowed to cool down naturally.

Finally, the sample (ZnO NRs/CuO composite film) was cleaned ultrasonically in ethanol and deionized water (DI water) for 30 min for several times and then dried at 60 ^o^C for 12 h in an oven under the atmospheric conditions to remove organic residuals and evaporate the remained DI water.

For comparison purpose, the ZnO NRs film, CuO film, and CuO/ZnO NR composite film on glass substrates were also fabricated by the same process for each films.

### Characterization

The crystal phases of the fabricated samples were determined using an X-ray diffractometer (XRD) D5000 with CuKα radiation (λ = 1.5406 Å) over the 2θ range 30~70° at room temperature. The surface morphologies were characterized using a field emission scanning electron microscopy (FESEM, Hitachi, S-4800). The optical absorption spectrum was measured by a UV-Vis spectrophotometer (Jasco, V-670).

### Photocatalytic activity measurement

The photocatalytic activity measurement was carried out at room temperature. In this work, a 250 W Xenon lamp was used as a light source and was placed about 30 cm vertically relative to the organic pollution solution to diminish the heat effect. The organic pollution solution temperature was kept at 27 ^o^C by the circulating cool water. The fabricated samples (2 cm × 2 cm) were immersed in 100 ml RhB solution with the initial concentration of 10 ppm under stirring and maintained in the dark for 30 min to allow adsorption-desorption equilibrium before light irradiation. The schematic diagram of photocatalytic activity measurement was shown in Fig. S1. During the photocatalytic activity measurement, after each given interval (20 min), 3 mL of solution was withdrawn and analyzed by an UV-vis spectrometer (Jasco, V-670) with RhB peak at a wavelength of 554 nm. For the reusability test of the ZnO NRs/CuO composite film in photocatalytic activity, after each cycle, the composite film was rinsed to remove residual molecules and re-immersed into a fresh RhB solution with the same volume and concentration. The process was repeated for 3 times to confirm the reusability of the ZnO NRs/CuO composite film as a photocatalyst. The degradation efficiency of RhB molecules was calculated from the following equation:

1 $$ \% Degradation=\frac{{C}_{o}-{C}_{t}}{{C}_{o}}\times 100 \% $$

where C_o_ and C_t_ are the initial absorbance and the absorbance at a certain time t, respectively.

### The simulation program SCAPS

In this work, the simulation program SCAPS (Solar Cell Simulation Program in One Dimension) was utilized to investigate the ZnO NRs/CuO composite film. This program has been normally designed to simulate some kind of thin film solar cells such as CIGS, CdTe, and etc^35–37^. Based on this program some characteristics like I-V, C-V, C-f, QE(λ), etc. can be calculated according to the variation in the parameters of materials and operating conditions. Furthermore, the photocatalytic activity strongly depends on the number of the photo-generated electron-hole pairs which can reach to the counter parts and do the degradation work. These active electron-hole pairs also relate to the photon induced current density. Therefore, this simulation program SCAPS can be utilized to find out the opto-electrical characteristics of the ZnO NRs/CuO composite film. The simulation results were also compared to experimental results and find out optimal parameters of the ZnO NRs/CuO composite film (see Supplementary Information).

## Supplementary information

Supplementary information.
