# Supplementary material for: Experimental and numerical study on photocatalytic activity of the ZnO nanorods/CuO composite film
Source: Sci Rep. 2020 May 8;10:7792. doi: 10.1038/s41598-020-64784-w (PMC7211011; doi:10.1038/s41598-020-64784-w)
Supplement: Supplementary file 1 — Supplementary information. [file 41598_2020_64784_MOESM1_ESM.docx]

Supplementary Information:

**Experimental and numerical study photocatalytic activity of the ZnO nanorods/CuO composite film**

Dung T. Nguyen^1^, Tran Minh Duc^1^, Hoang Van Thanh^1^, Trinh Duc Thien^2^, Pham Duc Thang^1^, Nguyen Nang Dinh^1^, and Nguyen Dinh Lam^1,*^

^1^Faculty of Engineering Physics and Nanotechnology, VNU University of Engineering and Technology, Vietnam National University, 144 Xuan Thuy Road, Cau Giay District, Hanoi, Vietnam

^2^Faculty of Physics, Hanoi National University of Education, 136 Xuan Thuy Road, Cau Giay District, Hanoi, Vietnam

### *Corresponding Author’s Email: [***lamnd2005@gmail.com***](mailto:lamnd2005@gmail.com)

1. **Photocatalytic activity measurement**

The photocatalytic activity measurement was carried out at room temperature. In this work, a 250 W Xenon lamp was used as a light source and was placed about 30 cm vertically relative to the organic pollution solution to diminish the heat effect. The organic pollution solution temperature was kept at 27 ^o^C by the circulating cool water. The fabricated samples (2 cm x 2 cm) were immersed in 100 mL RhB solution with the initial concentration of 10 ppm under stirring and maintained in the dark for 30 min to allow adsorption-desorption equilibrium before light irradiation.The schematic diagram of photocatalytic activity measurement was shown in Figure S1

1. **The simulation program SCAPS**

Poisson, the continuity equation of holes, and the continuity equation of electrons equations were numerically solved by SCAPS software for the electrostatic potentials of electrons and hole concentrations as a function of positions x.

Poisson’s equation is given as:

$\frac{d^{2}}{{dx}^{2}}\psi\left( x \right)= \frac{e}{\varepsilon_{o}\varepsilon_{r}}\left[ n\left( x \right)-p\left( x \right)-N_{D}^{+}\left( x \right)+N_{A}^{-}\left( x \right)-p_{t}\left( x \right)+n_{t}(x) \right]$ (1)
where ψ is electrostatic potential, ε_o_ and ε_r_ are the vacuum and relative permittivity constant, e is an electrical charge, p, and n are hole and electron concentrations, respectively, $N_{D}^{+}$is the ionized donor-like doping density, $N_{A}^{-}$is the ionized acceptor-like doping density, p_t_ is the trapped hole density, and n_t_ is the trapped electron density.

And the continuity equations define the transportation of carriers:

$\left\{ \begin{matrix} e\frac{\partial n}{\partial t}=\frac{{\partial J}_{n}}{\partial x}+eG-eR \\ e\frac{\partial p}{\partial t}=-\frac{{\partial J}_{p}}{\partial x}+eG-eR \end{matrix} \right.$ (2)

$\left\{ \begin{matrix} J_{n}=en\mu_{n}\frac{\partial\psi}{\partial x}+eD_{n}\frac{\partial n}{\partial x} \\ J_{p}=-en\mu_{p}\frac{\partial\psi}{\partial x}+eD_{p}\frac{\partial p}{\partial x} \end{matrix} \right.$ (3)

where G is the optical generation rate, R is the recombination rate, D_n_ is the electron diffusion coefficient, D_p_ is the hole diffusion coefficient, μ_n_ and μ_p_ are the electron and hole mobility, respectively.

A schematic structure of ZnO NRs/CuO heterojunction is shown in Figure S2. The material parameters sourced from experiments and used in this simulation were given in Table S1.

For investigation of the influences of the light illumination on different sides of ZnO NRs/CuO composite film, the thickness of CuO, ZnO layer and donor density in the ZnO NRs layer were kept as constants of 300 nm, 300 nm, and 1x10^16^ cm^-3^, respectively.

For investigation of the influences of the thickness of the CuO layer, the thickness of the ZnO layer and donor density in the ZnO NRs layer is kept as constants of 300 nm, and 1x10^16^ cm^-3^, respectively. The other parameters were also kept constant. J-V and quantum efficiency characteristics were generated based on the variation of the thickness of the CuO layer and depicted in Figure S3. The influence of the thickness of the CuO on the short circuit current density was extracted and shown in Figure S4. The short circuit current density reaches to saturation state when the thickness of the CuO layer is over 1000 nm. This can be contributedto the optical absorption efficiency of the thicker CuO layer.

For investigation the influences of the thickness of the ZnO NRs layer, the thickness of the CuO layer and donor density in the ZnO NRs layer is kept as constants of 1000 nm, and 1x10^16^ cm^-3^, respectively. The other parameters were also kept constant. J-V and quantum efficiency characteristics were generated based on the variation of the thickness of the ZnO layer and depicted in Figure S5. The influence of the thickness of the ZnO on the short circuit current density was extracted and shown in Figure S6. The short circuit current density is reduced as increasing in the thickness of the ZnO NRs layer. This can be attributed to the shallow penetration of UV light which is absorbed by the ZnO NRs layer.

For investigation of the influences of the donor density in the ZnO NRs layer, the thickness of the CuO and ZnO NRs layers were kept as constants of 1000 nm and 10 nm, respectively. The other parameters were also kept constant. J-V and quantum efficiency characteristics were generated based on the variation of the thickness of the ZnO layer and depicted in Figure S7. The donor density in the ZnO NRs layer slightly influences the optoelectric of the ZnO NRs/CuO heterojunction and rarely affects when the donor density is lower than 10^16^ cm^-3^. This result indicated that, for photocatalyst application using the ZnO NRs/CuO composite film, the dopant in ZnO film lacks efficiency.

Supplementary **Table S1**

| Parameter | n-ZnO | p-CuO |
| --- | --- | --- |
| Thickness, w (µm) | Varied | Varied |
| Bandgap, E_g_ (eV) | 3.3 | 1.51 |
| Electron affinity, χ (eV) | 4.4 | 4.07 |
| Dielectric constant, ε/ε_o_ | 9 | 18.10 |
| Density of states at conduction band, N_C_ (cm^-3^) | 3.1x10^18^ | 2.2x10^19^ |
| Density of states at valance band, N_V_ (cm^-3^) | 1.7x10^19^ | 5.5x10^20^ |
| Electron thermal velocity, δ_e_ (cm/s) | 1x10^7^ | 1x10^7^ |
| Hole thermal velocity, δ_h_ (cm/s) | 1x10^7^ | 1x10^7^ |
| Donor density, N_D_ (cm^-3^) | Varied | 0 |
| Acceptor density, N_A_ (cm^-3^) | 0 | 1x10^16^ |
| Electron mobility, μ_e_ (cm^2^/Vs) | 100 | 100 |
| Hole mobility, μ_h_ (cm^2^/Vs) | 25 | 0.10 |


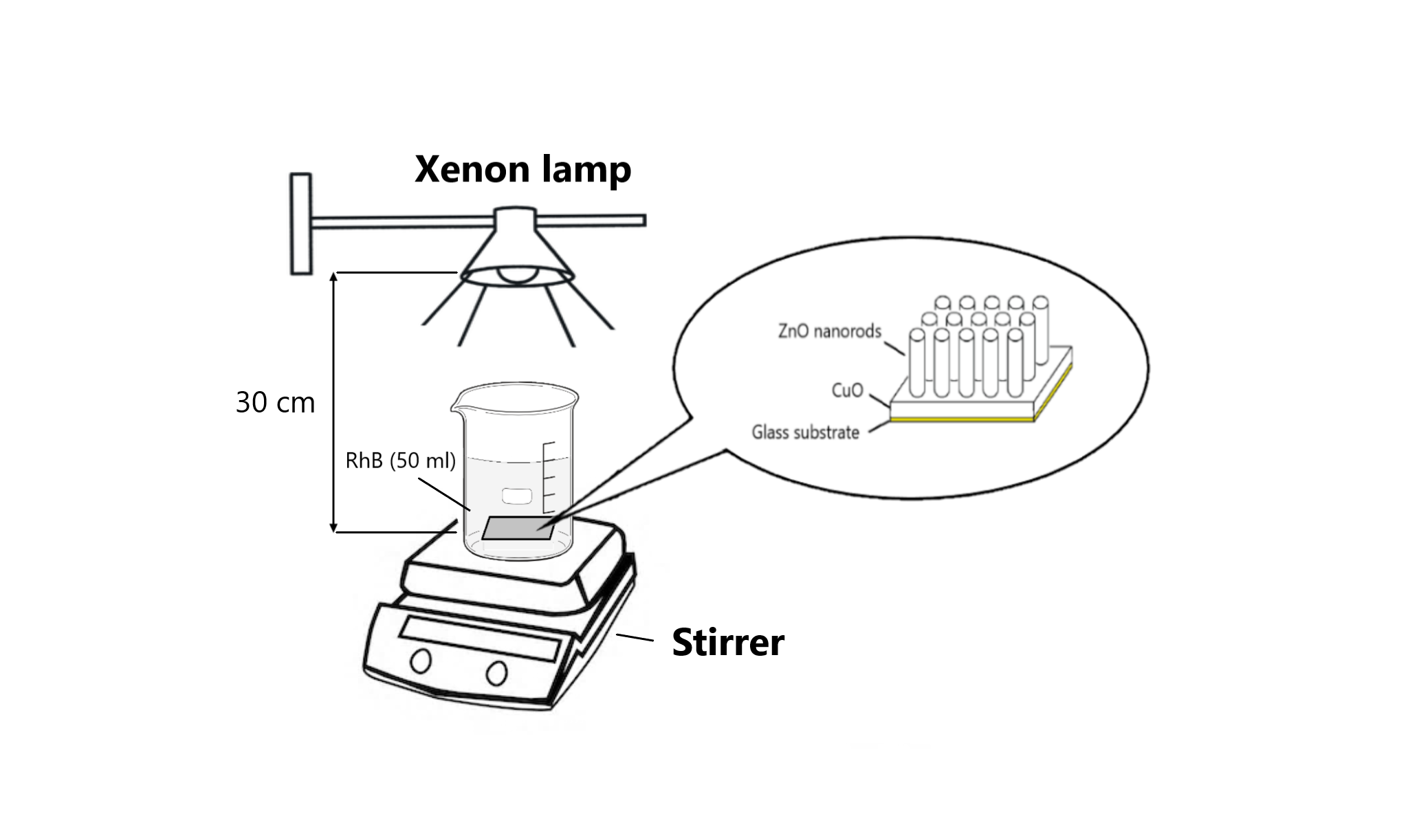


Supplementary **Figure S1:** The schematic diagram of photocatalytic activity measurement


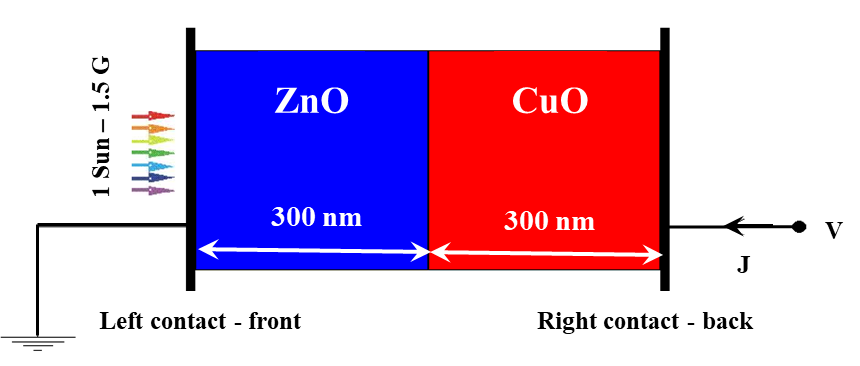


Supplementary **Figure S2:** Schematic structure of ZnO/CuO heterojunction

a

****b

Supplementary **Figure S3**: (a)J-V curves and (b) Quantum efficiencies of the ZnO/CuO heterojunction with different p-CuO layers in thickness. The thickness and N_D_ of the ZnO layer were kept at 300 nm and 1x10^16^ cm^-3^, respectively.

Supplementary **Figure S4: S**hort circuit current density related to the thickness of the CuO layer.

a

**b**

Supplementary **Figure S5**: (a) J-V curves and (b) Quantum efficiencies of the ZnO/CuO heterojunction with different n-ZnO layers in thickness. The thickness of the CuO layer and N_D_ of the ZnO layer was kept at 1 μm and 1x10^16^ cm^-3^, respectively.

Supplementary **Figure S6: S**hort circuit current density related to the thickness of the ZnO layer.

**a**

**b**

Supplementary **Figure S7**: (a) J-V curves and (b) Quantum efficiencies of the ZnO/CuO heterojunction with different donor density in the ZnO layer. The thickness of CuO and ZnO layers were kept at 1 μm and 10 nm, respectively.
